# Machine Learning with Social Determinants of Health for Cross-Sectional Diabetes Classification: Development and Temporal Validation

**DOI:** 10.7150/ijms.137605

**Published:** 2026-09-11

**Authors:** Woon-Man Kung, Hsien Chang Yeh, Abel Po-Hao Huang, Md. Mohaimenul Islam, Chieh-Chen Wu, Yao-Chin Wang

**Affiliations:** 1 Division of Neurosurgery, Department of Surgery, Taipei Tzu Chi Hospital, Buddhist Tzu Chi Medical Foundation, New Taipei City 23142, Taiwan.; 2 Interdisciplinary Program Kinesiology and Health, Chinese Culture University, Taipei 11114, Taiwan.; 3 School of Medicine, College of Medicine, Taipei Medical University, Taipei 11031, Taiwan.; 4 Division of Neurosurgery, Department of Surgery, National Taiwan University Hospital, College of Medicine, National Taiwan University, Taipei 10617, Taiwan.; 5 Division of Outcomes and Practice Advancement, Department of Pharmacy Practice, School of Pharmacy and Pharmaceutical Sciences, University at Buffalo, Buffalo, New York 14214, United States.; 6 Department of Healthcare Information and Management, School of Health and Medical Engineering, Ming Chuan University, Taoyuan 33300, Taiwan.; 7 Department of Emergency Medicine, Taipei Medical University Hospital, Taipei 11030, Taiwan.; 8 Graduate Institute of Injury Prevention and Control, College of Public Health, Taipei Medical University, Taipei 11031, Taiwan.

**Keywords:** type 2 diabetes, risk prediction, database research, disease prevention

## Abstract

**Aims:**

Our study aimed to determine whether incorporating social determinants of health (SDOH) improves cross-sectional classification of prevalent diagnosed diabetes and to quantify the extent to which model performance is attributable to variables that may occur downstream of diabetes diagnosis.

**Methods:**

We analyzed data from the 2023 Behavioral Risk Factor Surveillance System (BRFSS) as the development cohort and the 2024 BRFSS as an independent temporal validation cohort. Analyses were restricted to adults aged 35 years or older residing in states that administered the SDOH module. The outcome was self-reported diagnosed diabetes. Before model development, 30% of the 2023 sample was reserved as a test set. Imputation, encoding, feature processing, and hyperparameter tuning were performed within nested cross-validation. Histogram-based gradient boosting and penalized logistic regression were evaluated across six pre-specified nested predictor specifications. Final models were applied unchanged to the 2024 cohort without refitting, re-tuning, or threshold recalibration. Performance estimates incorporated BRFSS survey weights.

**Results:**

The development cohort comprised 179,752 adults (weighted prevalence 16.97%) and the validation cohort 161,092 (18.07%). Discrimination ranged from 0.75 to 0.81 across algorithms, with gradient boosting and penalized logistic regression indistinguishable (0.811 versus 0.808). The full model achieved an area under the receiver operating characteristic curve (AUROC) of 0.811 (95% CI 0.801 - 0.822) and transported to the validation cohort with a calibration slope of 1.040. Adding nine SDOH items changed discrimination by 0.006 (95% CI 0.004 - 0.008), with maximum net benefit gain under 0.003; discrimination rose from 0.743 to 0.811 only when healthcare utilization and comorbidity measures were added. Several SDOH nonetheless showed robust adjusted associations, including food insecurity (OR 1.27 - 1.39) and supplemental nutrition assistance program receipt (OR 1.34, 95% CI 1.11 - 1.61). At a fixed threshold, sensitivity ranged from 0.62 to 0.89 across age groups.

**Conclusions:**

SDOH were independently associated with prevalent diagnosed diabetes but provided little incremental value beyond demographic, behavioral, and healthcare access measures, a finding that was consistent across consecutive survey cycles. Most of the observed discrimination was attributable to healthcare utilization and comorbidity variables that may plausibly reflect consequences of an established diagnosis rather than characteristics preceding disease recognition.

## Introduction

Diabetes is a major and growing global health burden, affecting an estimated 589 million adults worldwide, or approximately one in nine, with prevalence projected to reach 853 million by 2050 1, 2. In 2024, diabetes was associated with 3.4 million deaths and more than one trillion dollars in health expenditures 3. Yet an estimated 252 million adults with diabetes remain undiagnosed 4, highlighting an important opportunity for earlier identification and prevention of downstream complications. Accurate risk stratification is central to that effort.

Most diabetes risk prediction research has emphasized established clinical and behavioral factors, including adiposity, physical inactivity, and family history 5, 6. Social determinants of health (SDOH) have received comparatively less attention, despite evidence linking food insecurity, housing instability, low income, and lower educational attainment with diabetes risk and poorer glycemic outcomes 7, 8. These conditions also contribute to persistent racial, ethnic, and socioeconomic disparities in diabetes burden, and their integration into screening and prevention strategies has been increasingly advocated 8. Recent machine learning studies have therefore begun incorporating SDOH alongside conventional predictors, with several reporting high discriminatory performance 9, 10.

However, the clinical and methodological significance of these performance estimates remains uncertain. Many studies emphasize discrimination while providing limited assessment of calibration, clinical utility, or transportability across populations or time periods 11. External and temporal validation remain uncommon, and complex survey sampling is not consistently incorporated into model development or evaluation. An additional concern is temporal ambiguity in cross-sectional data. When predictors and diabetes status are measured contemporaneously, variables such as recent healthcare use and diagnosed comorbidities may partly reflect consequences of an established diagnosis rather than characteristics that preceded it 12, 13. Models that derive substantial predictive performance from such variables may therefore distinguish individuals with recognized disease more effectively than they identify those whose diabetes is not yet clinically apparent 14, 15. Whether SDOH provides meaningful predictive information beyond demographic, behavioral, and healthcare access characteristics under these conditions remains unclear.

Using two consecutive cycles of the Behavioral Risk Factor Surveillance System (BRFSS), we evaluated the incremental contribution of SDOH to the classification of prevalent diagnosed diabetes beyond demographic, behavioral, and healthcare access factors. We further examined the extent to which model performance was attributable to variables that may plausibly occur downstream of diabetes diagnosis and assessed temporal transportability by developing models in one survey cycle and applying them without modification to the subsequent cycle.

## Methods

### Study design and data source

We conducted a cross-sectional classification study using two consecutive cycles of the BRFSS, an annual telephone survey of non-institutionalized adults conducted by the Centers for Disease Control and Prevention across all states and participating territories^16^. The 2023 BRFSS served as the development cohort, and the 2024 BRFSS served as an independent temporal validation cohort. Because predictors and diabetes status were ascertained during the same interview, the analysis was designed to classify prevalent diagnosed diabetes rather than predict incident disease. Accordingly, temporal ordering between candidate predictors and diabetes diagnosis could not be established.

### Outcome ascertainment

The primary outcome was prevalent self-reported clinician-diagnosed diabetes, ascertained from the BRFSS core chronic conditions section. Respondents were asked, *“Has a doctor, nurse, or other health professional ever told you that you had diabetes?”* Female respondents answering affirmatively were asked whether the diagnosis occurred only during pregnancy, and prediabetes or borderline diabetes was recorded separately when volunteered. The corresponding diabetes variable distinguishes six responses: diagnosed diabetes, gestational diabetes only, no diabetes, prediabetes or borderline diabetes, uncertain, and refused.

Respondents reporting diagnosed diabetes were classified as cases and those reporting no diabetes as non-cases. Three response categories were excluded to preserve an unambiguous case definition. Gestational diabetes only was excluded because it denotes a pregnancy-specific condition distinct from persistent hyperglycemia. Prediabetes or borderline diabetes were excluded because it identifies an intermediate glycemic state rather than diabetes. Uncertain or refused responses were excluded because glycemic status could not be determined. Since the outcome reflects a report of prior diagnosis rather than a laboratory measurement, the analysis addresses classification of diagnosed disease rather than detection of undiagnosed hyperglycemia, and self-report does not permit differentiation of diabetes subtype.

### Study population

Respondents were eligible if they were 35 years or older, resided in a state that administered the optional SDOH module, had a valid response to the diabetes outcome, and had a positive final survey weight. We restricted the study population to adults aged 35 years or older to focus on an age range in which prevalent diagnosed type 2 diabetes is more common and in which cumulative social and behavioral exposures are more likely to be relevant.

Because the SDOH module is optional and administered only in participating states, eligible module respondents were analyzed as a survey domain while retaining the complete BRFSS sampling design. This approach preserved the original strata and primary sampling units for variance estimation rather than treating module respondents as an independently sampled subpopulation.

### Pre-specified research questions

Three questions were specified before analysis: a) Does social determinants module improve classification of prevalent diabetes beyond demographic, financial access and behavioral measures? b) How much of the apparent discrimination of such models is attributable to healthcare utilization and comorbidity measures, which in cross-sectional data may reflect consequences of diagnosis rather than antecedent risk? and c) Do these findings replicate when a model developed in one survey cycle is applied without modification to the next?

### Predictors and model specifications

Candidate predictors were assigned a priori to six conceptual domains: demographics, SDOH, financial access to care, health behaviors, healthcare utilization, and comorbidity or health status. Variables were harmonized across the 2023 and 2024 BRFSS using the corresponding annual codebooks. Predictors unavailable in either survey cycle were excluded from all model specifications to ensure identical variable definitions during temporal validation. Standard BRFSS responses indicating “don't know/not sure” or refusal were coded as missing.

Six nested model specifications were pre-specified before model fitting. Model zero (M0) included age and sex only. Model one (M1) included the nine SDOH measures only (the nine SDOH measures are specified in **Supplementary Table S1**). Model two (M2) combined demographic characteristics and SDOH. Model three (M3) additionally incorporated financial access and health behaviors. Model four (M4) extended M3 by adding healthcare utilization measures, and model five (M5) further incorporated comorbidity and health status measures. A pre-specified comparator model, M3a, contained the same demographic, financial access, and behavioral variables as M3 but excluded the SDOH block. Comparison of M3 with M3a was used to estimate the incremental classification value of SDOH.

Model three was designated as the primary model because its predictors were considered more plausibly antecedent to diabetes diagnosis than those introduced in subsequent specifications. Healthcare utilization variables added in M4 may partly reflect the clinical contact through which diabetes was detected, whereas several comorbidity and health status measures added in M5 may represent manifestations or consequences of established disease. Changes in performance across M3 through M5 were therefore used to assess the extent to which apparent discrimination depended on variables potentially downstream of diagnosis.

### Data processing

All preprocessing was performed within a single modeling pipeline, with each transformation estimated exclusively from the training data within each resampling fold to prevent information leakage from held-out observations. Categorical variables were one-hot encoded, with missing values retained as a separate category to preserve potential information contained in survey nonresponse. Continuous variables were imputed using the median estimated within the corresponding training fold, and a missingness indicator was included for each imputed variable. Predictor specifications were defined as a priori, and no outcome-guided variable selection was performed.

Oversampling was not used in the primary analysis. Because synthetic oversampling changes the outcome distribution in the training data, it can alter predicted probabilities and compromise probability calibration unless appropriately corrected. We therefore prioritized the original outcome distribution for the primary analyses, particularly because model evaluation included calibration and decision-analytic measures. The influence of synthetic minority oversampling was evaluated separately in sensitivity analyses.

### Model building

Before model fitting, 30% of the development cohort was randomly reserved as an internal test set using stratification by diabetes status and state and was not accessed during model development or model selection (**Figure 1**). All model development was conducted within the remaining 70% development sample.

Nine classification algorithms were evaluated: penalized logistic regression, decision tree, random forest, extremely randomized trees, AdaBoost, Gaussian naive Bayes, histogram-based gradient boosting, eXtreme Gradient Boosting (XGBoost), and Light Gradient Boosting Machine (LightGBM). These algorithms represented a range of modeling approaches, from a parametric linear classifier to nonlinear tree-based and boosted ensemble methods. All algorithms were evaluated using the full predictor specification and identical preprocessing procedures. Models were fitted without survey weights, and the complex survey design was accommodated at the evaluation stage rather than during training. Standard implementations of the algorithms used treat observation weights as frequency or importance weights within the loss function and provide no mechanism for representing stratification or clustering, so weighted fitting would alter the effective loss without constituting design-based estimation. Because the quantity of interest was the population-level performance of a fitted classifier rather than a population parameter of the fitting procedure, models were fitted on the unweighted sample, and all performance estimates incorporated the BRFSS final sampling weight, with confidence intervals obtained by resampling respondents within design strata.

Algorithm selection was based on fivefold cross-validated out-of-fold predictions generated exclusively within the development sample, thereby preserving the internal test set for final evaluation. Hyperparameters for the leading candidate algorithms were optimized using nested cross-validation with five outer folds and three inner folds. Within each outer fold, hyperparameter tuning was restricted to the corresponding training partition, and the selected configuration was then refitted using the complete outer training partition and evaluated on the held-out outer fold. This procedure ensured that observations used to estimate model performance did not inform hyperparameter selection. Algorithms not subjected to tuning were fitted using pre-specified standard parameter settings. The algorithm with the highest cross-validated discrimination was subsequently used for all seven predictor specifications to ensure that comparisons across specifications reflected differences in predictor information rather than differences in modeling algorithms.

The classification threshold was fixed at the value, achieving 80% weighted sensitivity in development out-of-fold predictions and was not revised thereafter. Once algorithm selection, hyperparameter tuning, and threshold determination were complete, all models were frozen: the fitted estimator, the preprocessing parameters estimated during training, including imputation values and encoder categories, and the classification threshold were saved without further modification. Frozen models were then applied to the validation cohort, such that every prediction in the validation cohort derived from parameters determined exclusively in development cohort. Transportability was assessed before recalibration and after two corrections, updating the intercept alone and full recalibration of intercept and slope, which together distinguish drift in outcome prevalence from loss of validity in the relationship between predictors and outcome.

### Statistical analysis

All estimates incorporated the BRFSS final sampling weight. Descriptive comparisons used Taylor linearized variance estimation, and confidence intervals for performance measures were obtained by bootstrap resampling of respondents within design strata. Group differences were summarized by standardized mean differences rather than significance tests, since at this sample size trivial differences attain conventional significance.

Discrimination was quantified by the area under the receiver operating characteristic curve (AUROC) and by average precision. Calibration was assessed by the calibration slope, calibration in the large, the observed to expected ratio, the Brier and scaled Brier scores, and the integrated calibration index with its median and ninetieth percentile, supplemented by flexible spline calibration curves. Clinical usefulness was evaluated by decision curve analysis across threshold probabilities from 0.02 to 0.70, with treat all and treat none as reference strategies. Performance was additionally examined within subgroups defined by age, sex, race and ethnicity, education, household income, insurance status and urban rural residence.

Adjusted associations between individual social determinants and prevalent diagnosed diabetes were estimated using survey weighted logistic regression, mutually adjusted for the remaining social determinants and for demographic covariates, with robust standard errors. Reference categories were specified explicitly rather than assigned by default ordering.

### Model explanation

SHapley Additive exPlanations (SHAP) values were computed for the selected model to describe the contribution of each predictor to individual predictions and, aggregated as mean absolute values, to overall model behavior. Contributions were summarized at the level of the original survey variables and by conceptual block. We emphasize that SHAP quantifies a feature's contribution to the predictions of a given model and does not indicate incremental predictive value relative to a model omitting that feature. The nested specifications above provide this comparison directly. Analyses were performed in Python 3 using scikit-learn, statsmodels, and pandas.

## Results

### Study population

The development cohort comprised 179,752 respondents aged 35 years or older in states fielding the Social Determinants module, of whom 31,200 reported diagnosed diabetes, a survey weighted prevalence of 16.97% (95% CI 16.58 - 17.36). The validation cohort comprised 161,092 respondents, of whom 29,200 reported diagnosed with diabetes, a weighted prevalence of 18.07% (95% CI 17.60 - 18.54). Derivation of both cohorts is summarized in **Figure 2**, and their characteristics in **Supplementary Table S1.**

The cohorts were closely comparable despite partial turnover in participating states, with six fielding the module in 2023 but not 2024 and four in 2024 but not 2023. Across 183 covariate categories, no standardized mean difference between cycles exceeded 0.10, indicating that any difference in performance between cohorts is unlikely to reflect shifts in case mix.

### Model performance in the development cohort

Across the nine algorithms evaluated using the full predictor specification, discrimination was similar for the principal regression and ensemble methods (**Figure 3**). In the development cohort, cross-validated AUROCs were 0.80 (95% CI 0.79 - 0.81) for gradient boosting, XGBoost, penalized logistic regression and LightGBM; 0.79 for random forest, extra trees, and AdaBoost; and lower for the single decision tree (0.77) and Gaussian naive Bayes (0.75). The same ordering held in the internal validation set, where the four leading algorithms all achieved 0.81 (95% CI 0.80 - 0.82). Histogram-based gradient boosting attained the highest point estimate and was carried forward, although its advantage over penalized logistic regression was not distinguishable from sampling variation.

Threshold-based performance, calibration, and the difference between apparent and validated discrimination are reported for all algorithms in **Table 1**. Optimism, defined as the difference between re-substitution and held out area under the curve, was smallest for penalized logistic regression and largest for the unconstrained tree-based methods, consistent with the greater capacity of those models to fit training data. At the fixed operating threshold, the leading algorithms achieved comparable sensitivity and specificity, and calibration slopes near unity.

Applying the best performing algorithm (gradient boosting) to each of the seven pre-specified specifications produced a strongly graded pattern (**Figure 4**). The nine social determinants items alone discriminated poorly, at 0.60 (95% CI 0.59 - 0.62), below age and sex alone at 0.65 (95% CI 0.64 - 0.66). Adding demographic covariates raised discrimination to 0.732, and the further addition of financial access and health behaviors produced the primary specification at 0.74 (95% CI 0.73 - 0.75). Discrimination increased to 0.759 once healthcare utilization measures entered the model, and to 0.81 (95% CI 0.80 - 0.82) with the addition of comorbidity measures. The two blocks plausibly downstream of diagnosis therefore accounted for 0.068 of the area under the curve, approximately two thirds of the difference between the primary and full specifications. Removing the social determinants block from the primary specification changed discrimination by 0.001.

The full model was well calibrated in both cohorts (**Figure 5**). In the development test set the calibration slope was 1.064 with calibration in the large of 0.096 and an integrated calibration index of 0.013. In the validation cohort, the slope was 1.040, indicating that the relationship between predictors and outcome was essentially unchanged between cycles. The modest positive calibration in the large of 0.084 reflected the higher prevalence observed in 2024, and updating the intercept alone brought predicted and observed risks into close agreement.

Decision curve analysis showed the full model to exceed both default strategies of screening all and screening no one across threshold probabilities from 0.02 to approximately 0.60. At the pre-specified operating threshold of 0.131, net benefit was 0.105.

### Incremental value of social determinants

Removing the nine items from the primary specification reduced AUROC from 0.7429 to 0.7416, a difference of 0.0013 (**Figure 4**). Against a demographic model, the same block contributed 0.0059 (95% CI 0.0035 - 0.0080). Neither comparison indicated improved probability estimation: the scaled Brier score changed by 0.0009 and the integrated calibration index deteriorated slightly, from 0.0127 to 0.0134.

Decision curve analysis reinforced this finding (**Figure 5, panels C and D**). Across threshold probabilities from 0.02 to 0.70, the maximum gain in net benefit attributable to the social determinants block was 0.003, fewer than three additional true positives per thousand screened at any threshold.

### Temporal validation

Models were applied to the validation cohort without refitting, re-tuning or threshold adjustment. Discrimination was well preserved (**Figure 4**). The primary specification declined from 0.743 to 0.731 (95% CI 0.723 - 0.738) and the full specification from 0.811 to 0.804 (95% CI 0.799 - 0.810). Losses across all specifications ranged from 0.006 to 0.016.

Calibration behaved as expected under a modest shift in prevalence (**Figure 5, panels A and B**). The calibration slope of the full specification was 1.040 in validation, indicating that the predictor outcome relationship was essentially unchanged between cycles, while calibration in the large of 0.084 reflected the higher 2024 prevalence. Updating the intercept alone restored calibration completely, returning the observed to expected ratio to 1.000 and reducing the integrated calibration index from 0.0147 to 0.0057. Subsequent slope recalibration produced no material further improvement. The incremental contribution of the social determinants block replicated in the independent cycle, with discrimination improving by 0.0033 and maximum net benefit gain of 0.0022 across all thresholds.

### Adjusted associations

Although the block conferred negligible classification value, several individual measures showed robust adjusted associations in survey weighted logistic regression (**Supplementary Table S2**). Food insecurity displayed a graded association relative to respondents whose food always lasted, with odds ratios of 1.27 (95% CI 1.07 - 1.52) for rarely, 1.27 (95% CI 1.02 - 1.57) for sometimes, 1.32 (95% CI 0.95 - 1.83) for usually and 1.39 (95% CI 1.03 - 1.88) for always. Receipt of Supplemental Nutrition Assistance Program benefits was independently associated with the outcome at 1.34 (95% CI 1.11 - 1.61), and dissatisfaction with life at 1.52 (95% CI 1.14 - 2.03), although the latter is plausibly a consequence of living with diagnosed disease. Loneliness, emotional support, transport barriers, inability to pay bills and threatened utility disconnection showed no association after adjustment.

### Subgroup performance

Performance varied substantially across subgroups at the fixed operating threshold (**Figure 6; Supplementary Table S3**). For full specification, sensitivity ranged from 0.62 to 0.89 across age groups and calibration in the large varied by 0.60 across racial and ethnic categories. The primary specification showed wider variation in sensitivity, from 0.38 among respondents aged 40 to 44 years to 0.94 among those aged 75 to 79, with systematic under prediction among respondents with less than a high school education and among Hispanic respondents. Subgroup estimates were also less stable across cycles than aggregate performance, shifting by up to 0.076 while overall discrimination changed by no more than 0.016.

### Feature contributions

In the full model, SHAP attribution was concentrated in clinical and anthropometric measures rather than social conditions (**Figure 7**). Self-rated general health and body mass index category carried the largest mean absolute contributions, followed by age group and recency of routine checkup. Obesity and fair general health shifted predictions toward diabetes. Excellent or very good health and ages 35 to 44 years shifted them away. Diabetes complications, including coronary heart disease or myocardial infarction and kidney disease, contributed substantially when reported. Two measures of healthcare contact - a routine checkup within the past year and having a personal healthcare provider ranked among the ten most influential features, reproducing within the fitted model the dependence on healthcare utilization that the nested comparisons quantified. Social determinants ranked low, with food insecurity and emotional support the only such items among the 20 highest contributions, each accounting for less than one-tenth of the attribution assigned to general health. SHAP values describe how predictors influence the output of this model and do not quantify incremental predictive value relative to a model omitting them.

## Discussion

In two consecutive BRFSS cycles, SDOH were consistently associated with prevalent diagnosed diabetes but provided little incremental value for classification. Adding the SDOH module to models containing demographic, behavioral, and access-related characteristics produced minimal improvement in discrimination and no meaningful increase in net benefit across clinically relevant thresholds. Larger gains occurred only after inclusion of healthcare utilization and comorbidity measures. In addition, nine algorithms ranging from penalized logistic regression to boosted ensemble methods yielded similar discrimination. These findings suggest that model performance was driven primarily by predictor content rather than algorithmic complexity.

The distinction between association and predictive utility is important. Several SDOH measures, including food insecurity and receipt of nutrition assistance, were independently associated with prevalent diagnosed diabetes 17-19. However, these variables contributed little additional discrimination once demographic and socioeconomic characteristics were included. This finding is not contradictory. A factor may have a strong epidemiologic association with disease yet contribute little to classification when its information overlaps with variables already in the model or when its effect is insufficient to meaningfully separate individuals with and without the outcome 20, 21. Thus, the inclusion of SDOH in a high-performing model should not be interpreted as evidence that these factors are responsible for the model's discriminatory performance.

The largest improvement in discrimination followed the addition of healthcare utilization and comorbidity measures. Because these variables and diabetes status were measured concurrently, their temporal relation to diabetes cannot be established 22-25. Some may reflect greater contact with the healthcare system, recognition of established diabetes, consequences of chronic disease, or shared underlying risk 26, 27. Accordingly, a model using these variables may partly distinguish individuals whose diabetes has already been recognized rather than identify individuals before diagnosis. Such models may have value for surveillance or case ascertainment, but their performance should not be interpreted as evidence of prospective risk prediction. Reporting models that exclude variables susceptible to diagnostic or disease-related feedback may help distinguish prediction of antecedent risk from classification of established disease.

Our estimates were lower than those reported in some previous machine learning studies of diabetes 28-30, which may reflect differences in evaluation rather than differences in model quality. Class balancing through oversampling can alter the training outcome distribution and, without correction, affect probability calibration 31. Accuracy may also be misleading when outcome prevalence is low because high values can be achieved by favoring the majority class 32. In addition, preprocessing performed before resampling and threshold selection based on the same observations used for evaluation can introduce optimism 33. In the present study, preprocessing, model selection, tuning, threshold determination, internal testing, and temporal validation were separated to limit information leakage and provide a more stringent assessment of performance.

Performance also varied across population subgroups. At a single operating threshold, sensitivity differed by age, and calibration varied across racial and ethnic and educational groups, including underprediction among Hispanic adults and adults without a high school education. Subgroup performance was also less stable across survey cycles than overall performance. These findings indicate that acceptable aggregate discrimination and calibration do not ensure comparable performance across population groups. Evaluation before implementation should therefore include subgroup-specific discrimination, calibration, and threshold-based performance, particularly when models are intended to support equitable screening or case identification.

Temporal validation was comparatively reassuring. When applied without modification to the subsequent BRFSS cycle, the model retained similar discrimination and a calibration slope near one. The principal calibration difference was consistent with a change in outcome prevalence and improved after intercept updating. This pattern suggests relative stability in predictor-outcome associations across survey cycles. However, temporal transportability does not establish clinical usefulness. A model may remain well calibrated and discriminative over time while providing little incremental benefit or relying on variables that become informative only after disease recognition.

### Limitations

This study has several limitations. First, diabetes status was based on self-reported clinician diagnosis rather than laboratory measurement, so adults with undiagnosed diabetes were classified as not having diabetes, and the models addressed classification of diagnosed disease rather than detection of undiagnosed hyperglycemia. This also implies that predictors related to engagement with healthcare are associated with the outcome partly through their influence on whether disease is detected, reinforcing our interpretation of the healthcare utilization block. Second, self-report does not distinguish diabetes subtype. Restriction to adults aged 35 years or older reduces but does not eliminate type 1 diabetes among cases, and because these determinants are not hypothesized to operate similarly for type 1 diabetes, such misclassification would attenuate rather than inflate the associations reported. Third, the cross-sectional design precluded determination of temporal ordering and does not permit inference regarding incident diabetes or causality. Fourth, the optional SDOH module was administered only in participating states, so the analytic sample does not represent all US states and territories, and some respondents completed multi-version questionnaires for which version-specific weighting may be relevant. Fifth, the available SDOH measures capture only selected dimensions of social disadvantage and omit neighborhood, environmental, structural, and longitudinal exposures. Sixth, we did not examine weighted model fitting, although the narrow range of discrimination across methods makes a substantial effect unlikely. Seventh, two clinically relevant rotating-core variables were available in the development cycle but not the validation cycle and could not enter temporally validated models. Finally, these findings pertain to a single survey, outcome, and cross-sectional classification setting and may not generalize to laboratory-confirmed diabetes, longitudinal risk prediction, or more granular SDOH measurement.

## Conclusions

In two consecutive BRFSS cycles, SDOH were associated with prevalent diagnosed diabetes but provided little incremental improvement in discrimination or clinical net benefit. More complex machine learning algorithms offered little advantage over penalized logistic regression, whereas the largest gains in performance followed inclusion of healthcare utilization and comorbidity measures that may partly reflect established disease or its recognition. These findings underscore that epidemiologic association does not necessarily confer predictive utility and that performance of cross-sectional classification models should be interpreted in relation to the variables from which that performance is derived.

## Supplementary Material

Supplementary tables.

## Figures and Tables

**Figure 1:**
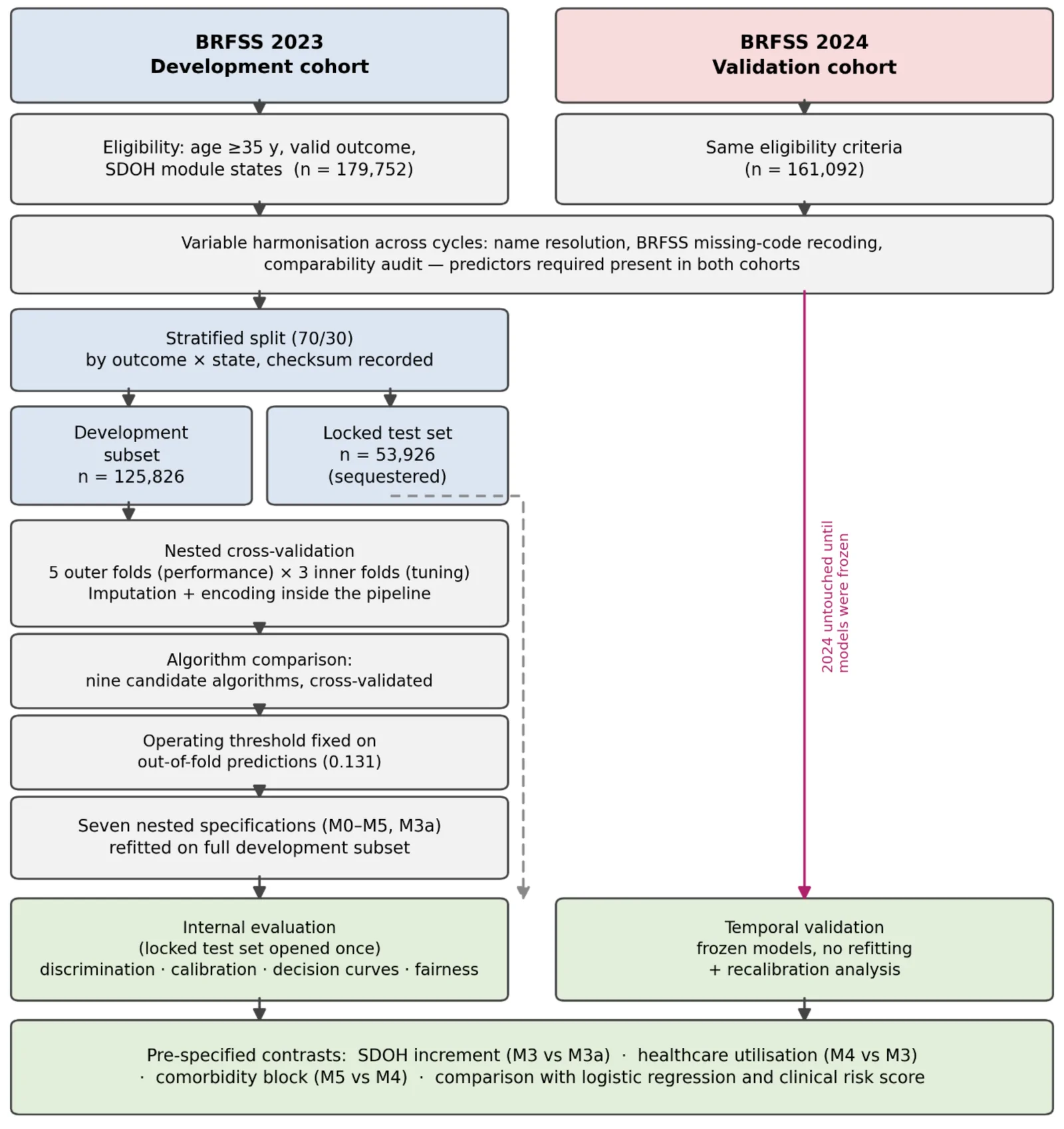
Study design and analysis workflow. Models were developed exclusively in the Behavioral Risk Factor Surveillance System (BRFSS) 2023 cohort and applied to the BRFSS 2024 cohort without refitting, re-tuning, or threshold adjustment.

**Figure 2:**
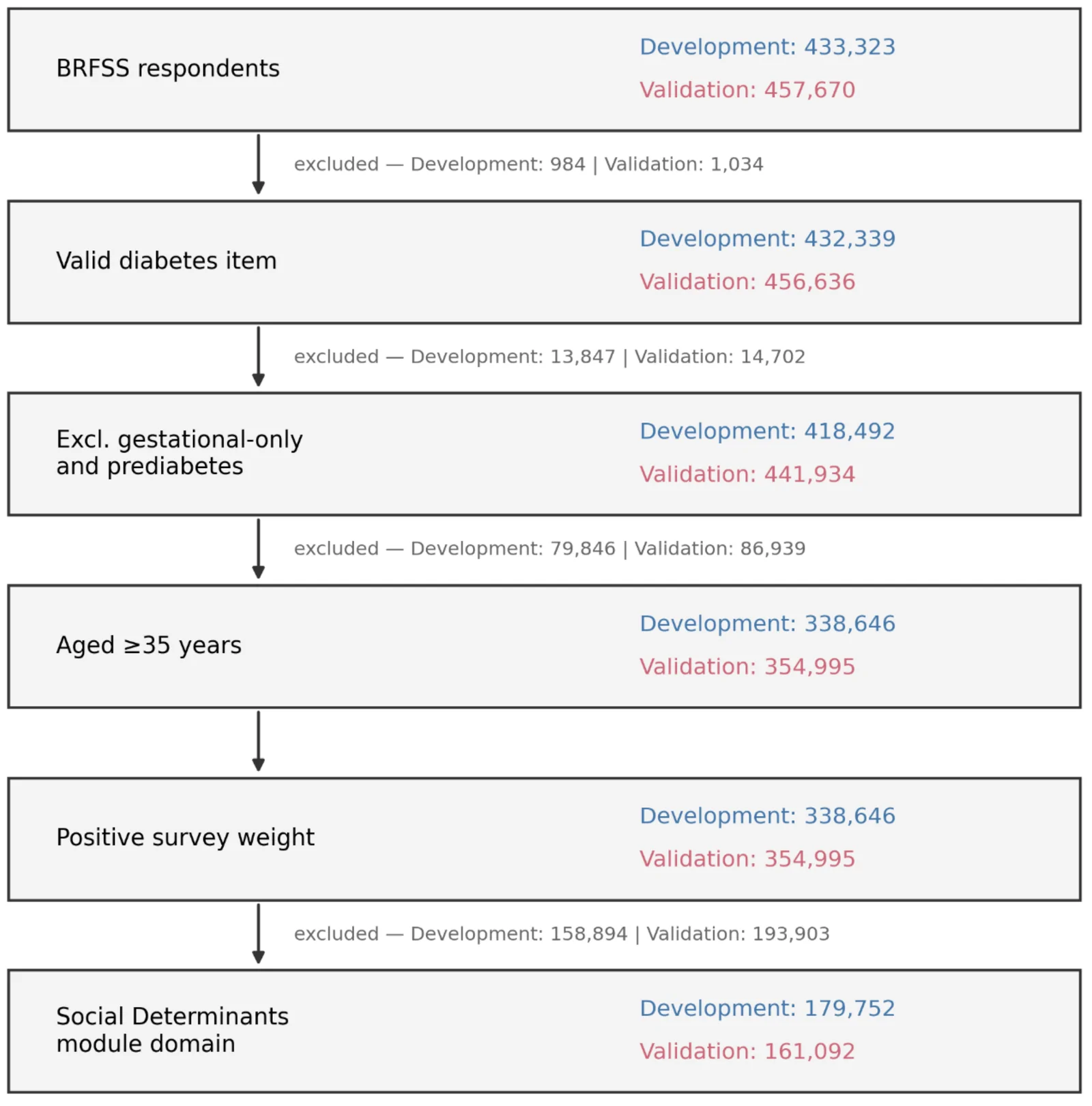
Derivation of the development and validation cohorts, Behavioral Risk Factor Surveillance System 2023 and 2024.

**Figure 3:**
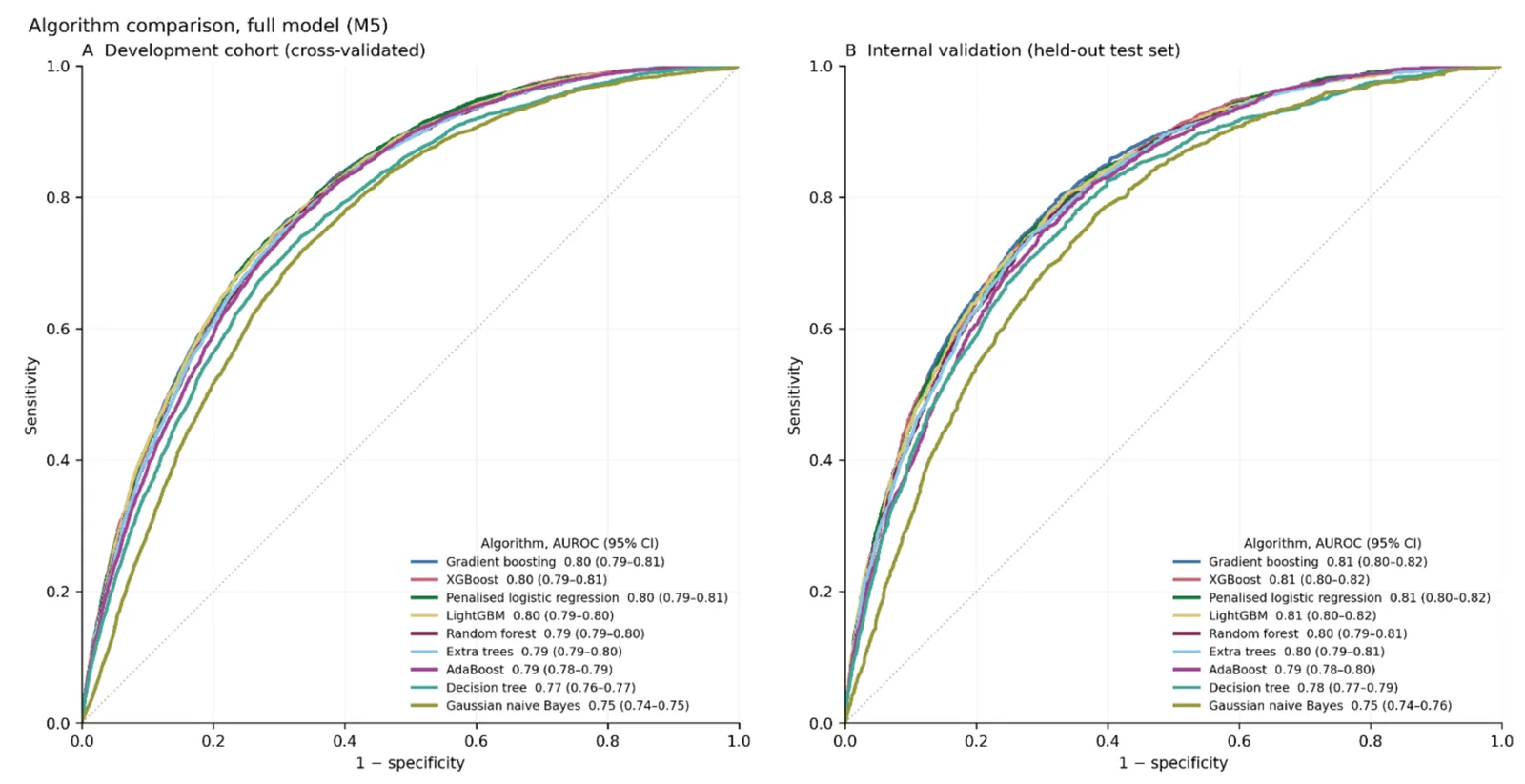
Discrimination of nine candidate algorithms applied to the full model, with cross-validated and internal validation estimates.

**Figure 4:**
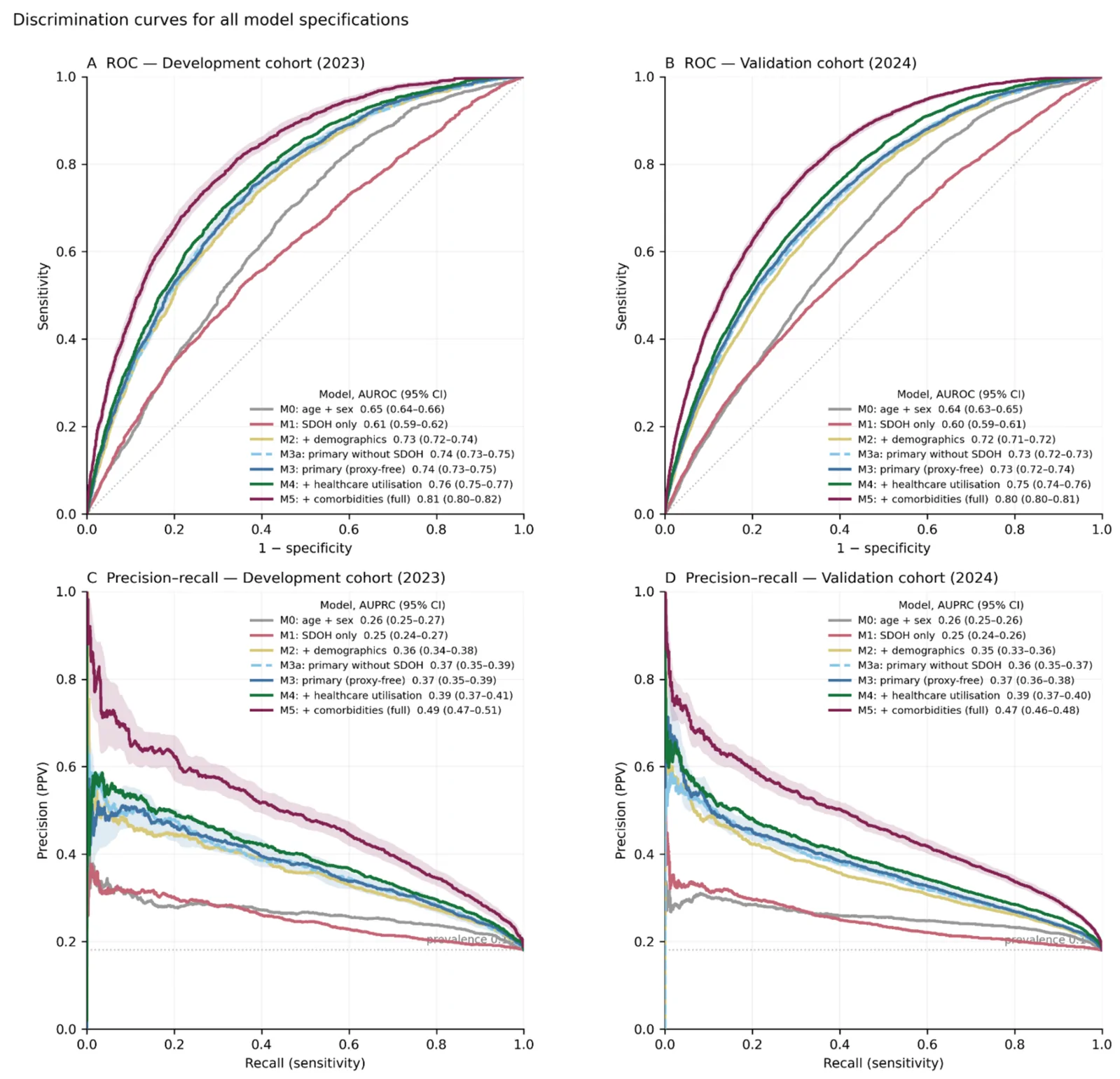
Discrimination across nested model specifications in the development and temporal validation cohorts.

**Figure 5:**
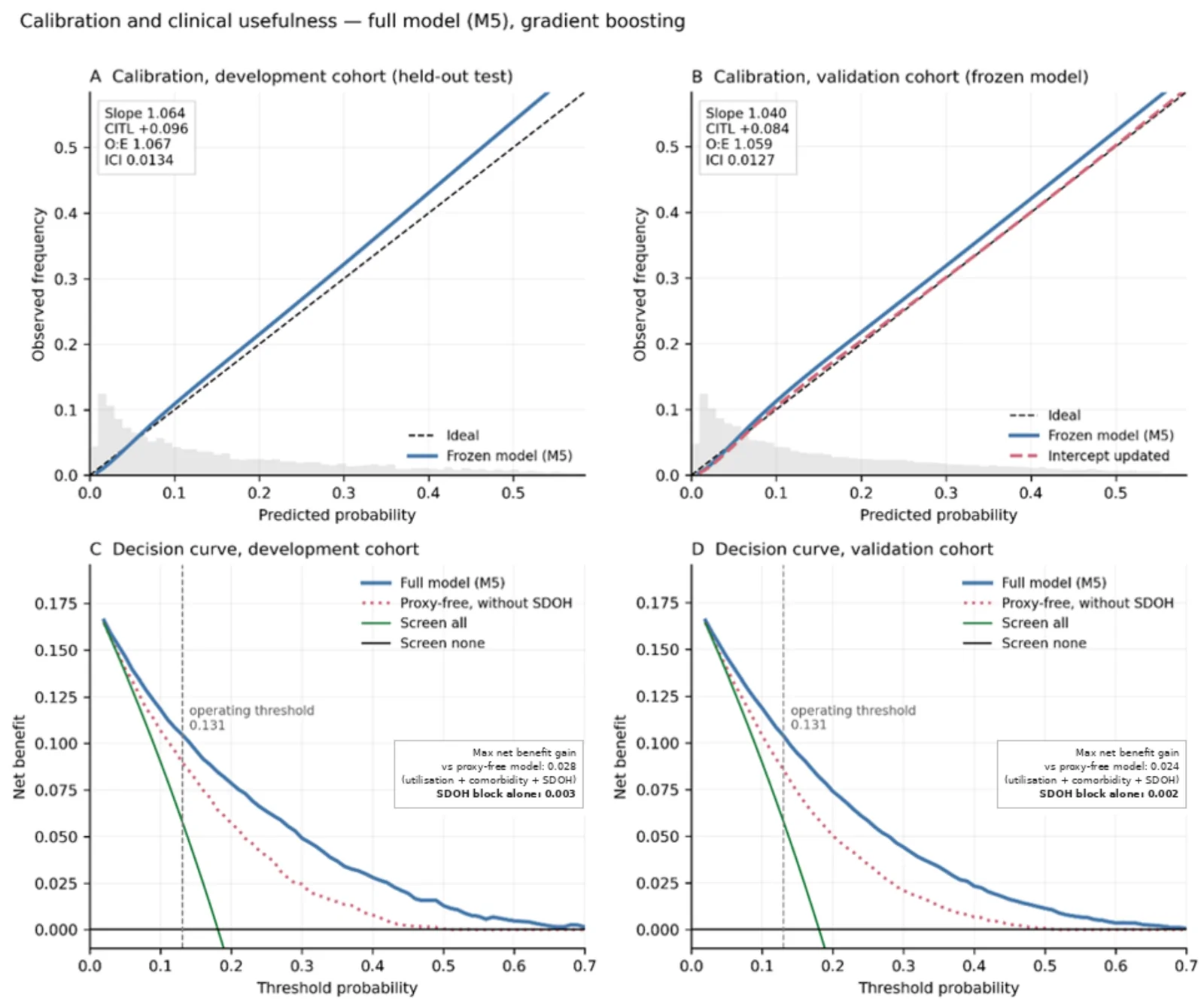
Calibration and decision curve analysis for the full model in both cohorts.

**Figure 6:**
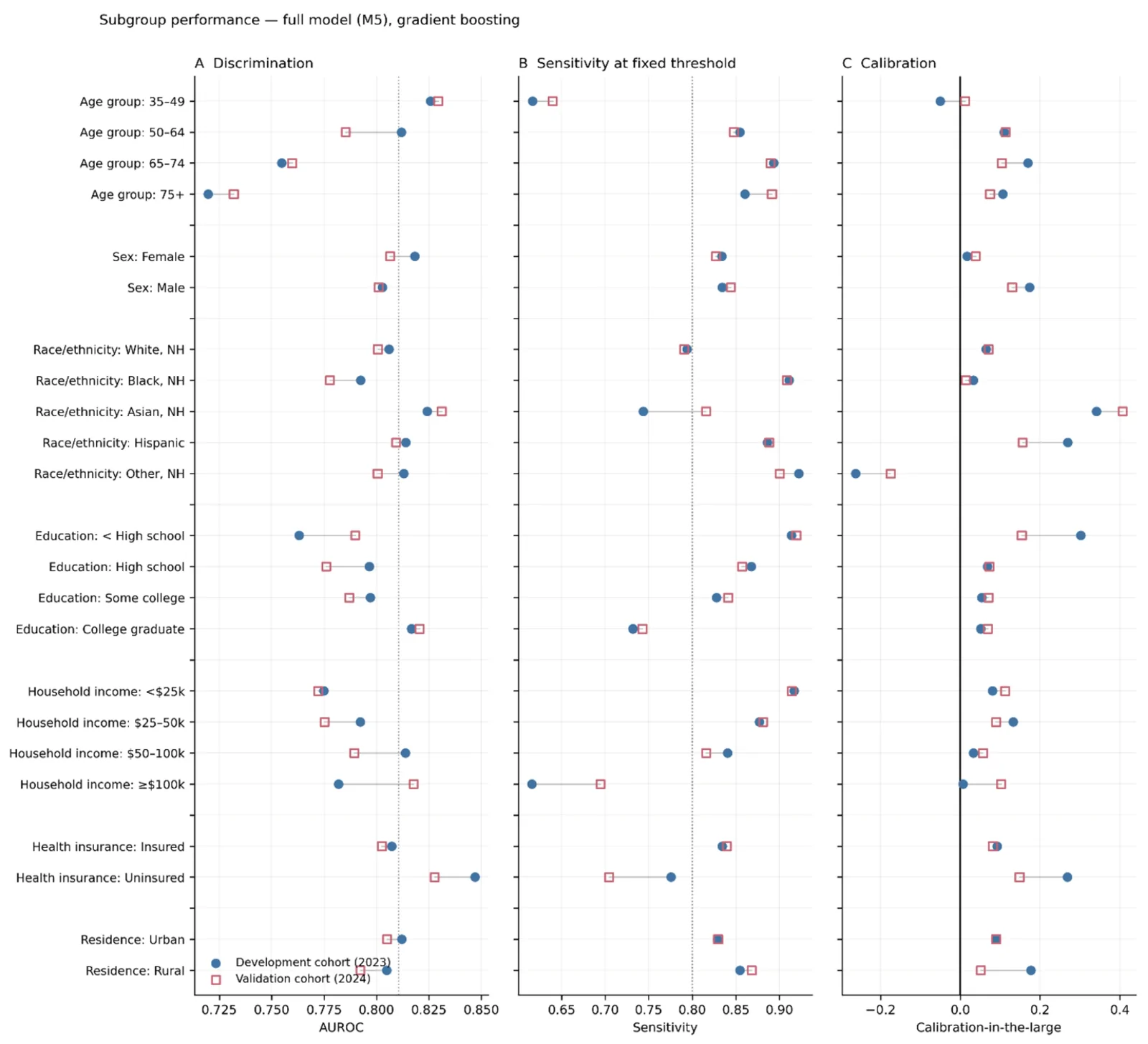
Subgroup performance of the full model at the fixed operating threshold in both cohorts.

**Figure 7:**
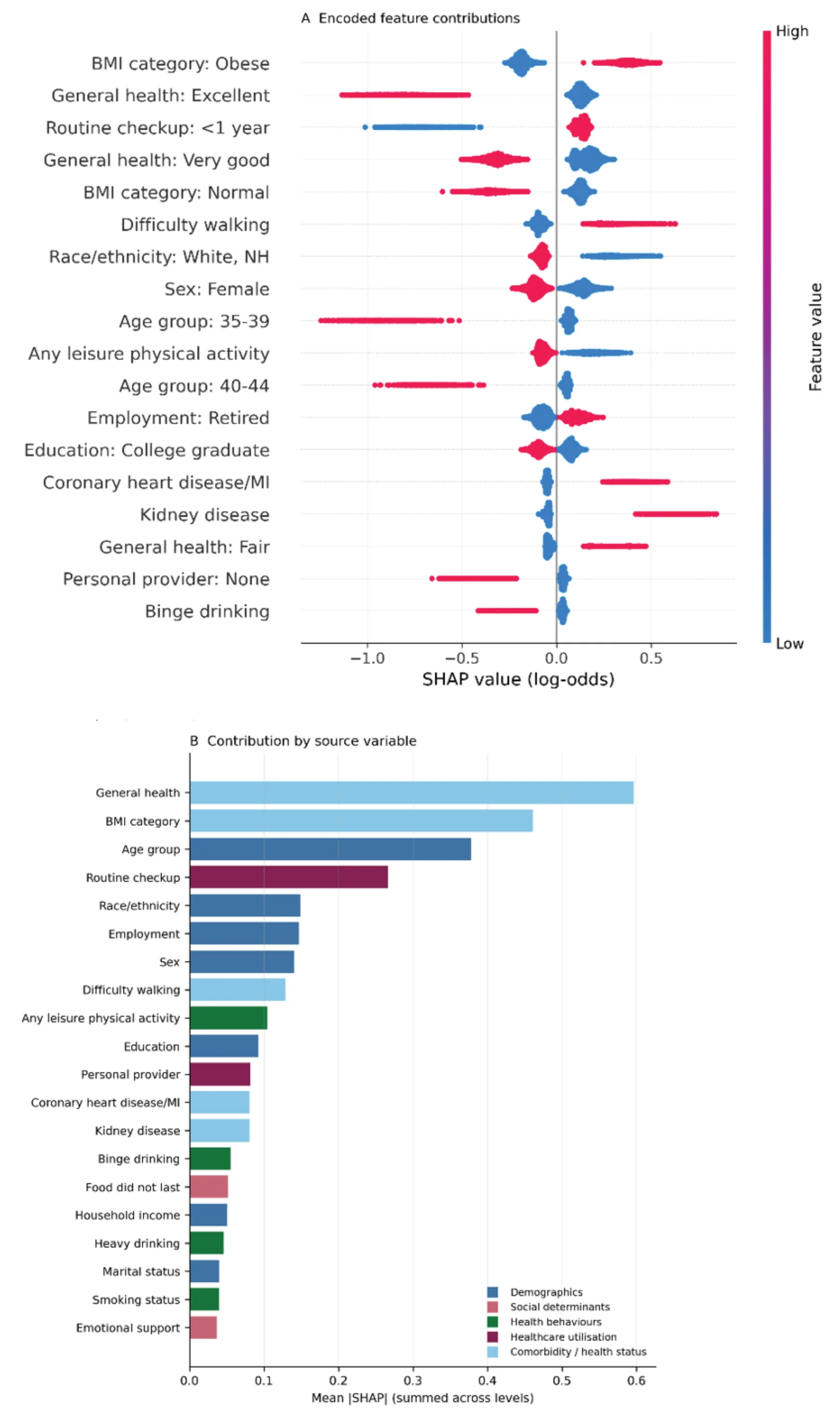
SHapley Additive exPlanations feature contributions for the full model in the development cohort test set.

**Table 1:** Performance of nine candidate algorithms applied to the full model in the internal validation cohort.

Algorithm	AUROC internal validation (95% CI)	AUPRC	Sensitivity	Specificity	F1 score	Brier	Calibration slope	ICI
Gradient boosting	0.81 (0.80 - 0.82)	0.48	0.83	0.63	0.47	0.119	1.06	0.013
XGBoost	0.81 (0.80 - 0.82)	0.48	0.83	0.63	0.47	0.119	0.95	0.012
Penalized logistic regression	0.81 (0.80 - 0.82)	0.48	0.83	0.62	0.47	0.119	1.04	0.013
LightGBM	0.81 (0.80 - 0.82)	0.48	0.82	0.63	0.47	0.119	0.96	0.010
Random forest	0.80 (0.79 - 0.81)	0.47	0.85	0.58	0.45	0.121	1.15	0.016
Extra trees	0.80 (0.79 - 0.81)	0.47	0.85	0.58	0.46	0.121	1.09	0.013
AdaBoost	0.79 (0.78 - 0.80)	0.45	1.00	0.53	0.31	0.169	4.29	0.199
Decision tree	0.78 (0.77 - 0.79)	0.44	0.83	0.58	0.45	0.125	0.99	0.013
Gaussian naive Bayes	0.75 (0.74 - 0.76)	0.36	0.63	0.74	0.45	0.252	0.08	0.250

AUROC = area under the receiver operating characteristic curve; CI = confidence interval; AUPRC = area under the precision-recall curve; ICI = integrated calibration index; XGBoost = eXtreme Gradient Boosting; LightGBM = Light Gradient Boosting Machine; AdaBoost = Adaptive Boosting Note: AdaBoost derives predicted probabilities from a rescaled exponential loss, producing values that are strongly compressed toward the center of the range. At the fixed operating threshold of 0.131, applied uniformly across algorithms, nearly all respondents fell above this cutoff, yielding sensitivity of 1.00 with correspondingly low positive predictive value and F1 score. The calibration slope of 4.29 reflects this compression of the predicted probability distribution rather than a failure of discrimination, which was comparable to that of other ensemble methods (cross-validated AUROC 0.79). Threshold-based metrics for this algorithm should therefore be interpreted because of applying a common threshold to an uncalibrated probability scale.

## Data Availability

Data will be made available on request.
