# Supplementary material for: Machine Learning with Social Determinants of Health for Cross-Sectional Diabetes Classification: Development and Temporal Validation
Source: Int J Med Sci. 2026 Sep 11;23(11):3323–35. doi: 10.7150/ijms.137605 (PMC13685035; doi:10.7150/ijms.137605)
Supplement: Supplementary file 1 [file ijmsv23p3323s1.pdf]

Supplementary Table S1. Baseline characteristics.

Characteristic	Level	2023 Diabetes	2023 No diabetes	2023 SMD	2024 Diabetes	2024 No diabetes	2024 SMD	Cross-cycle SMD
Total, n (weighted prevalence %)		31,200	149,810		29,200	132,522		
Weighted prevalence of diabetes, %		17.66			18.07			
Age group	35-39	595 (3.0)	12,451 (12.1)	0.352	529 (2.5)	11,332 (12.3)	0.383	0.001
	40-44	902 (4.6)	12,850 (13.2)	0.307	956 (5.6)	11,983 (13.8)	0.279	0.018
	45-49	1,433 (5.8)	11,900 (9.8)	0.148	1,398 (7.3)	10,867 (10.4)	0.112	0.028
	50-54	2,229 (10.1)	13,433 (11.7)	0.054	2,109 (10.4)	11,233 (10.8)	0.014	0.023
	55-59	2,952 (11.2)	14,310 (10.0)	0.037	2,744 (11.6)	12,229 (9.7)	0.061	0.006
	60-64	4,209 (16.3)	17,224 (11.9)	0.125	3,840 (15.1)	14,960 (11.7)	0.102	0.012
	65-69	5,160 (14.1)	19,201 (9.5)	0.143	4,764 (14.4)	16,471 (9.7)	0.147	0.007
	70-74	5,243 (14.3)	17,756 (8.6)	0.18	4,843 (13.4)	15,495 (8.2)	0.166	0.015
	75-79	4,383 (10.6)	13,969 (5.9)	0.171	4,188 (10.4)	12,765 (6.4)	0.147	0.014
	80+	4,094 (10.1)	16,716 (7.2)	0.104	3,829 (9.4)	15,187 (7.0)	0.085	0.009
Sex	Female	16,078 (49.9)	82,516 (53.2)	0.067	14,803 (48.5)	71,995 (52.2)	0.074	0.022
	Male	15,122 (50.1)	67,294 (46.8)	0.067	14,397 (51.5)	60,527 (47.8)	0.074	0.022
Race Ethnicity	White NH	22,119 (54.4)	119,024 (64.6)	0.209	20,454 (52.7)	103,853 (62.9)	0.207	0.038
	AI/AN NH	751 (1.4)	2,169 (1.0)	0.031	758 (1.4)	1,995 (1.1)	0.027	0.005
	Asian NH	671 (5.3)	3,128 (5.5)	0.01	559 (6.2)	2,573 (5.4)	0.037	0.001
	Black NH	3,489 (15.6)	9,646 (10.4)	0.155	3,636 (15.4)	9,745 (10.8)	0.135	0.009
	Hispanic	3,078 (19.7)	11,381 (14.7)	0.134	2,824 (20.8)	10,346 (16.0)	0.125	0.034
	Other NH	1,092 (3.5)	4,462 (3.7)	0.008	969 (3.5)	4,010 (3.9)	0.018	0.009
Education	Missing	84 (0.2)	379 (0.2)	0.006	85 (0.4)	321 (0.2)	0.023	0.007
	<High school	2,750 (18.4)	6,986 (9.7)	0.25	2,379 (17.1)	6,086 (9.7)	0.219	0.006
	High school	8,804 (27.3)	32,683 (23.1)	0.096	8,181 (26.7)	29,134 (22.6)	0.096	0.012
	Some college	9,422 (31.3)	39,425 (30.6)	0.015	8,782 (32.4)	34,422 (30.2)	0.047	0.001
	College graduate	10,140 (22.8)	70,337 (36.4)	0.3	9,773 (23.4)	62,559 (37.2)	0.305	0.016

Household income	Missing	5,646 (19.8)	24,748 (16.4)	0.086	4,716 (18.4)	20,469 (16.0)	0.063	0.016
	<$15k	2,301 (7.9)	5,903 (4.3)	0.152	1,917 (6.9)	5,071 (4.2)	0.122	0.013
	$15 – 25k	3,575 (11.5)	9,991 (6.7)	0.169	3,270 (11.4)	8,525 (6.2)	0.183	0.015
	$25 – 35k	3,813 (11.6)	12,548 (7.8)	0.128	3,534 (11.9)	11,008 (8.0)	0.131	0.007
	$35 – 50k	4,159 (12.4)	16,253 (10.2)	0.068	3,863 (11.4)	13,976 (9.5)	0.061	0.026
	$50 – 100k	7,253 (21.0)	39,257 (23.5)	0.061	7,222 (21.8)	34,684 (23.6)	0.044	0.005
	$100 – 200k	3,605 (11.8)	29,956 (21.6)	0.265	3,775 (14.1)	27,984 (21.9)	0.206	0.015
	>$200k	848 (3.9)	11,154 (9.3)	0.218	903 (4.2)	10,805 (10.6)	0.247	0.037
Marital status	Missing	127 (0.4)	769 (0.5)	0.028	120 (0.4)	587 (0.5)	0.004	0.006
	Divorced	4,965 (13.4)	21,398 (12.4)	0.03	4,700 (14.1)	19,123 (13.0)	0.032	0.019
	Married	15,673 (54.6)	86,491 (61.4)	0.139	14,666 (55.9)	76,159 (60.7)	0.099	0.008
	Never married	3,341 (10.8)	15,132 (10.9)	0.002	3,199 (10.4)	13,731 (11.3)	0.028	0.009
	Separated	709 (3.3)	2,714 (2.5)	0.047	671 (3.2)	2,368 (2.3)	0.053	0.009
	Unmarried couple	687 (2.4)	4,461 (3.3)	0.056	648 (2.6)	4,137 (3.7)	0.061	0.018
	Widowed	5,698 (15.2)	18,845 (9.0)	0.191	5,196 (13.3)	16,417 (8.5)	0.155	0.024
Employment	Missing	162 (0.8)	929 (0.8)	0.002	182 (0.7)	855 (0.9)	0.022	0.013
	Employed	7,213 (27.0)	57,982 (45.6)	0.392	7,115 (29.5)	51,586 (45.5)	0.335	0.007
	Homemaker	1,094 (4.8)	6,094 (5.3)	0.022	991 (4.6)	5,066 (5.0)	0.018	0.013
	Out of work 1 year	538 (2.0)	2,248 (2.0)	0.003	527 (2.6)	2,048 (2.1)	0.034	0.016
	Out of work <1 year	406 (1.7)	2,264 (2.0)	0.025	418 (2.2)	2,141 (2.2)	0.002	0.02
	Retired	16,063 (43.1)	57,567 (27.5)	0.33	14,695 (39.4)	50,817 (27.1)	0.264	0.02
	Self-employed	1,581 (5.8)	14,458 (10.8)	0.182	1,506 (6.1)	12,826 (11.0)	0.177	0.007
	Student	44 (0.1)	477 (0.4)	0.061	59 (0.2)	420 (0.5)	0.044	0.007
	Unable to work	4,099 (14.7)	7,791 (5.7)	0.301	3,707 (14.6)	6,763 (5.7)	0.3	0.002
Home ownership	Missing	145 (0.6)	732 (0.6)	0.001	126 (0.5)	536 (0.5)	0.004	0.003
	Own	23,372 (77.3)	120,385 (81.8)	0.111	21,923 (78.0)	105,811 (81.9)	0.096	0.004
	Rent	6,477 (18.6)	24,758 (15.5)	0.084	6,054 (18.2)	22,694 (15.5)	0.073	0.002
	Other	1,206 (3.5)	3,935 (2.2)	0.078	1,097 (3.3)	3,481 (2.1)	0.07	0.005

Veteran	Missing	47 (0.1)	244 (0.2)	0.018	43 (0.1)	190 (0.2)	0.007	0.007
	No	25,919 (86.2)	131,093 (89.4)	0.096	24,367 (86.8)	116,024 (89.4)	0.079	0.004
	Yes	5,234 (13.7)	18,473 (10.5)	0.099	4,790 (13.1)	16,308 (10.5)	0.081	0.003
Rural Urban	Missing	820 (3.4)	2,678 (2.0)	0.088	958 (3.7)	3,118 (2.4)	0.073	0.026
	Rural	5,234 (9.4)	22,389 (7.7)	0.06	4,166 (7.9)	17,803 (7.1)	0.03	0.027
	Urban	25,146 (87.2)	124,743 (90.3)	0.099	24,076 (88.4)	111,601 (90.4)	0.066	0.011
Health insurance	Missing	966 (2.9)	3,931 (2.6)	0.021	851 (3.1)	3,193 (2.6)	0.028	0.003
	No	704 (3.5)	5,725 (5.3)	0.085	663 (3.6)	5,280 (5.5)	0.091	0.01
	Yes	29,530 (93.5)	140,154 (92.1)	0.054	27,686 (93.3)	124,049 (91.9)	0.055	0.01
Cost barrier to care	Missing	88 (0.4)	392 (0.3)	0.012	102 (0.4)	343 (0.3)	0.026	0.003
	No	28,786 (90.4)	139,971 (91.8)	0.049	26,749 (89.8)	122,950 (90.9)	0.039	0.028
	Yes	2,326 (9.3)	9,447 (7.9)	0.047	2,349 (9.8)	9,229 (8.8)	0.034	0.029
Personal provider	Missing	182 (0.6)	992 (0.7)	0.01	169 (1.0)	934 (0.8)	0.015	0.024
	None	1,024 (4.0)	13,993 (11.3)	0.275	939 (4.2)	12,406 (12.2)	0.293	0.023
	1	17,062 (54.7)	85,755 (56.0)	0.025	15,711 (54.1)	74,439 (54.8)	0.014	0.023
	>1	12,932 (40.7)	49,070 (32.1)	0.179	12,381 (40.8)	44,743 (32.3)	0.177	0.004
Routine checkup	Missing	236 (1.0)	1,454 (1.0)	0.002	198 (0.6)	1,152 (1.0)	0.045	0.011
	Never	47 (0.3)	718 (0.7)	0.061	47 (0.2)	633 (0.7)	0.076	0
	<1 year	29,555 (93.5)	123,793 (79.7)	0.415	27,786 (94.3)	109,760 (79.8)	0.443	0.007
	1-2 years	901 (3.3)	12,020 (9.2)	0.248	762 (3.1)	10,793 (9.1)	0.251	0.006
	2-5 years	292 (1.3)	6,073 (5.0)	0.215	245 (1.0)	5,110 (5.0)	0.238	0.002
	>5 years	169 (0.6)	5,752 (4.3)	0.238	162 (0.8)	5,074 (4.4)	0.228	0.002
General health	Missing	113 (0.4)	370 (0.2)	0.023	99 (0.5)	308 (0.2)	0.041	0.007
	Poor	3,715 (12.9)	5,835 (3.8)	0.332	3,353 (12.7)	5,085 (3.7)	0.333	0.003
	Fair	8,898 (31.6)	18,642 (13.1)	0.456	8,347 (30.6)	16,575 (13.1)	0.434	0.004
	Good	11,998 (36.1)	47,800 (32.6)	0.075	11,433 (37.3)	43,508 (33.4)	0.081	0.019
	Very good	5,458 (15.4)	53,610 (34.0)	0.44	5,097 (15.9)	46,774 (33.2)	0.41	0.013
	Excellent	1,018 (3.6)	23,553 (16.3)	0.435	871 (3.0)	20,272 (16.3)	0.465	0.005
Poor physical health days	Missing	1,035 (3.3)	3,229 (2.0)	0.078	980 (3.5)	2,715 (1.9)	0.097	0.004
	0 days	14,445 (47.0)	93,083 (63.1)	0.328	13,485 (47.4)	81,774 (62.6)	0.31	0.008

	1 - 13 days	7,816 (24.4)	34,689 (22.8)	0.039	7,412 (24.3)	31,174 (23.3)	0.024	0.01
	>14 days	7,904 (25.3)	18,809 (12.1)	0.343	7,323 (24.8)	16,859 (12.2)	0.33	0.001
Poor mental health days	Missing	666 (2.1)	2,443 (1.6)	0.033	589 (2.2)	1,967 (1.6)	0.042	0.003
	0 days	19,397 (62.1)	96,690 (62.7)	0.013	18,066 (60.8)	84,963 (62.3)	0.03	0.012
	1 - 13 days	6,355 (19.7)	34,473 (24.0)	0.104	6,141 (21.8)	31,256 (24.3)	0.059	0.013
	>14 days	4,782 (16.1)	16,204 (11.6)	0.13	4,404 (15.2)	14,336 (11.8)	0.098	0
Difficulty walking	Missing	173 (0.6)	485 (0.3)	0.046	151 (0.4)	481 (0.3)	0.025	0.008
	No	19,654 (63.3)	126,251 (85.7)	0.53	18,526 (64.9)	112,009 (86.1)	0.509	0.015
	Yes	11,373 (36.1)	23,074 (14.0)	0.526	10,523 (34.6)	20,032 (13.6)	0.508	0.014
Cognitive difficulty	Missing	237 (0.7)	825 (0.5)	0.017	211 (0.8)	678 (0.5)	0.039	0
	No	26,008 (82.6)	134,530 (89.3)	0.193	24,369 (82.5)	119,055 (89.3)	0.197	0.001
	Yes	4,955 (16.8)	14,455 (10.2)	0.193	4,620 (16.7)	12,789 (10.2)	0.192	0.001
BMI category	Missing	1,701 (5.9)	7,954 (5.8)	0.004	1,483 (5.4)	6,653 (5.5)	0.003	0.012
	Underweight	189 (0.7)	2,334 (1.4)	0.065	171 (0.6)	2,072 (1.5)	0.082	0.005
	Normal	4,225 (13.4)	43,696 (27.2)	0.348	4,067 (13.7)	38,048 (27.3)	0.34	0.001
	Obese	15,608 (49.5)	42,656 (30.1)	0.405	14,509 (49.7)	38,623 (29.9)	0.414	0.002
	Overweight	9,477 (30.5)	53,170 (35.6)	0.108	8,970 (30.5)	47,126 (35.9)	0.115	0.005
Coronary heart disease MI	Missing	563 (2.1)	1,344 (0.7)	0.114	532 (2.5)	1,243 (0.9)	0.123	0.018
	No	24,258 (79.3)	136,554 (92.6)	0.392	22,298 (78.5)	119,831 (92.3)	0.398	0.016
	Yes	6,379 (18.7)	11,912 (6.6)	0.368	6,370 (19.1)	11,448 (6.8)	0.37	0.01
Stroke	Missing	130 (0.4)	425 (0.2)	0.032	113 (0.3)	306 (0.2)	0.033	0.018
	No	27,982 (90.4)	143,336 (96.2)	0.233	26,082 (89.6)	126,564 (96.3)	0.263	0.005
	Yes	3,088 (9.1)	6,049 (3.5)	0.231	3,005 (10.1)	5,652 (3.6)	0.261	0.009
Asthma	Missing	128 (0.4)	412 (0.2)	0.032	116 (0.3)	384 (0.3)	0.001	0.001
	No	25,294 (80.9)	129,383 (86.6)	0.154	23,377 (80.4)	113,775 (85.8)	0.146	0.02
	Yes	5,778 (18.7)	20,015 (13.2)	0.15	5,707 (19.4)	18,363 (13.9)	0.147	0.021
Depressive disorder	Missing	162 (0.7)	603 (0.5)	0.031	155 (0.5)	521 (0.4)	0.019	0.019
	No	23,205 (75.3)	121,796 (82.2)	0.169	21,835 (76.2)	107,340 (81.4)	0.128	0.012
	Yes	7,833 (24.0)	27,411 (17.3)	0.165	7,210 (23.3)	24,661 (18.2)	0.126	0.015
COPD	Missing	181 (0.6)	549 (0.4)	0.036	178 (0.7)	483 (0.3)	0.058	0.001

	No	26,596 (86.7)	137,630 (92.8)	0.2	24,845 (86.6)	121,773 (93.1)	0.214	0.008
	Yes	4,423 (12.7)	11,631 (6.9)	0.196	4,177 (12.6)	10,266 (6.6)	0.205	0.008
Kidney disease	Missing	279 (1.0)	447 (0.3)	0.091	205 (1.2)	404 (0.3)	0.105	0.011
	No	26,493 (86.4)	143,397 (96.4)	0.36	24,661 (85.4)	126,480 (96.1)	0.376	0.02
	Yes	4,428 (12.6)	5,966 (3.4)	0.345	4,334 (13.5)	5,638 (3.6)	0.357	0.017
Arthritis	Missing	176 (0.6)	758 (0.5)	0.004	141 (0.8)	631 (0.5)	0.04	0.001
	No	14,447 (50.4)	94,449 (68.2)	0.367	12,979 (49.8)	81,541 (67.3)	0.362	0.019
	Yes	16,577 (49.0)	54,603 (31.3)	0.368	16,080 (49.4)	50,350 (32.2)	0.356	0.019
Smoking status	Missing	216 (0.5)	1,099 (0.8)	0.03	210 (0.7)	799 (0.6)	0.014	0.013
	Never	16,801 (56.0)	87,531 (59.6)	0.072	15,848 (56.4)	77,892 (60.0)	0.073	0.008
	Former	10,832 (31.9)	44,167 (27.2)	0.102	10,024 (32.4)	39,276 (27.9)	0.099	0.014
	Current, some days	875 (3.2)	4,573 (3.4)	0.013	781 (2.8)	4,041 (3.4)	0.031	0.007
	Current, daily	2,476 (8.3)	12,440 (8.9)	0.023	2,337 (7.6)	10,514 (8.2)	0.019	0.027
Current e-cigarette use	Missing	107 (0.5)	449 (0.4)	0.012	129 (0.4)	493 (0.4)	0.004	0.009
	No	30,340 (97.0)	144,272 (95.3)	0.085	28,302 (96.6)	127,409 (95.3)	0.064	0.005
	Yes	753 (2.5)	5,089 (4.2)	0.095	769 (3.0)	4,620 (4.3)	0.068	0.008
Smokeless tobacco	Missing	29 (0.1)	174 (0.1)	0.001	36 (0.1)	184 (0.2)	0.005	0.013
	No	30,359 (97.1)	145,756 (97.2)	0.007	28,344 (97.1)	128,843 (97.1)	0.004	0.004
	Yes	812 (2.8)	3,880 (2.7)	0.007	820 (2.8)	3,495 (2.7)	0.005	0.001
Any leisure physical activity	Missing	112 (0.4)	350 (0.2)	0.032	89 (0.2)	278 (0.2)	0.001	0.006
	No	12,735 (40.0)	34,985 (23.8)	0.355	10,947 (36.2)	28,058 (21.0)	0.342	0.067
	Yes	18,353 (59.6)	114,475 (76.1)	0.358	18,164 (63.6)	104,186 (78.8)	0.341	0.067
Heavy drinking	Missing	374 (1.3)	2,136 (1.7)	0.032	325 (1.4)	2,001 (1.7)	0.029	0.004
	No	30,075 (96.5)	138,124 (92.2)	0.185	28,108 (95.9)	122,092 (92.0)	0.165	0.008
	Yes	751 (2.2)	9,550 (6.1)	0.194	767 (2.7)	8,429 (6.2)	0.173	0.007
Binge drinking	Missing	366 (1.4)	2,274 (1.7)	0.026	355 (1.5)	2,128 (1.7)	0.02	0.002
	No	29,193 (92.8)	129,864 (84.9)	0.253	27,199 (91.5)	114,488 (84.6)	0.214	0.011
	Yes	1,641 (5.8)	17,672 (13.4)	0.26	1,646 (7.0)	15,906 (13.6)	0.219	0.012
Life satisfaction	Missing	378 (1.0)	1,269 (0.9)	0.01	299 (0.9)	981 (0.8)	0.004	0.008

	Very dissatisfied	511 (1.5)	1,495 (1.1)	0.037	509 (1.6)	1,366 (1.0)	0.05	0.001
	Dissatisfied	1,656 (5.4)	5,143 (3.4)	0.098	1,610 (4.8)	4,640 (3.5)	0.069	0.001
	Satisfied	16,446 (52.9)	68,878 (47.1)	0.115	15,483 (53.1)	61,019 (47.2)	0.118	0.002
	Very satisfied	12,209 (39.3)	73,025 (47.5)	0.167	11,299 (39.6)	64,516 (47.5)	0.159	0
Emotional support	Missing	569 (1.5)	2,330 (1.5)	0.001	522 (2.3)	1,980 (1.5)	0.054	0.017
	Never	1,557 (6.6)	5,081 (4.1)	0.11	1,588 (6.0)	5,137 (4.8)	0.056	0.02
	Rarely	1,365 (4.4)	4,818 (3.5)	0.044	1,440 (4.7)	4,873 (4.0)	0.034	0.023
	Sometimes	4,359 (14.7)	16,723 (11.9)	0.084	4,266 (14.1)	15,754 (12.9)	0.037	0.021
	Usually	7,735 (22.4)	44,739 (28.7)	0.143	7,256 (24.1)	39,071 (27.8)	0.083	0.011
	Always	15,615 (50.4)	76,119 (50.3)	0.001	14,128 (48.7)	65,707 (49.1)	0.007	0.026
Loneliness	Missing	307 (0.8)	1,227 (0.9)	0.01	223 (1.0)	957 (0.8)	0.024	0.004
	Never	12,810 (42.8)	63,839 (43.4)	0.011	11,428 (41.2)	53,973 (41.9)	0.015	0.03
	Rarely	8,106 (23.4)	46,881 (29.7)	0.143	8,024 (24.8)	43,459 (31.4)	0.148	0.036
	Sometimes	7,646 (25.2)	30,580 (20.7)	0.108	7,337 (24.9)	27,907 (20.6)	0.102	0.002
	Usually	1,163 (3.5)	4,071 (2.8)	0.043	1,143 (4.3)	3,621 (3.0)	0.074	0.017
	Always	1,168 (4.2)	3,212 (2.6)	0.091	1,045 (3.8)	2,605 (2.3)	0.09	0.021
SNAP receipt	Missing	252 (0.9)	1,115 (0.8)	0.016	206 (0.9)	910 (0.8)	0.01	0.002
	No	26,424 (82.9)	136,693 (89.6)	0.197	25,007 (84.0)	121,411 (90.1)	0.183	0.019
	Yes	4,524 (16.2)	12,002 (9.6)	0.198	3,987 (15.1)	10,201 (9.1)	0.186	0.019
Food did not last	Missing	392 (1.2)	1,701 (1.2)	0.003	337 (1.3)	1,280 (1.2)	0.014	0.001
	Never	22,836 (70.1)	124,555 (80.3)	0.236	21,350 (69.1)	109,664 (79.7)	0.245	0.015
	Rarely	2,804 (9.5)	9,820 (7.1)	0.088	2,757 (9.7)	9,022 (7.6)	0.075	0.019
	Sometimes	3,198 (12.1)	8,595 (7.3)	0.163	2,958 (12.8)	7,783 (7.3)	0.185	0.006
	Usually	857 (3.2)	2,146 (1.7)	0.095	752 (3.0)	2,091 (1.8)	0.076	0.005
	Always	1,113 (3.9)	2,993 (2.5)	0.079	1,046 (4.0)	2,682 (2.3)	0.095	0.006
Transport barrier	Missing	393 (1.3)	1,857 (1.5)	0.012	321 (1.6)	1,461 (1.3)	0.022	0.005
	No	28,103 (89.7)	140,562 (92.9)	0.114	26,356 (88.7)	124,331 (93.1)	0.155	0
	Yes	2,704 (9.0)	7,391 (5.6)	0.129	2,523 (9.7)	6,730 (5.5)	0.158	0.003
Utility shut-off threat	Missing	352 (1.3)	1,615 (1.3)	0.002	301 (1.5)	1,310 (1.3)	0.022	0
	No	28,504 (90.4)	140,417 (92.5)	0.073	26,634 (89.2)	124,134 (92.2)	0.102	0.016

	Yes	2,344 (8.3)	7,778 (6.3)	0.078	2,265 (9.3)	7,078 (6.6)	0.1	0.017
Unable to pay bills	Missing	372 (1.2)	1,557 (1.2)	0.007	327 (1.5)	1,267 (1.2)	0.03	0.006
	No	27,499 (85.5)	137,596 (89.6)	0.122	25,657 (84.4)	121,454 (89.2)	0.142	0.018
	Yes	3,329 (13.2)	10,657 (9.3)	0.125	3,216 (14.1)	9,801 (9.7)	0.138	0.016
Lost employment hours	Missing	488 (2.0)	1,650 (1.2)	0.07	399 (1.8)	1,283 (1.1)	0.057	0.003
	No	28,776 (89.8)	137,564 (89.6)	0.008	26,851 (89.4)	121,595 (89.6)	0.005	0.002
	Yes	1,936 (8.1)	10,596 (9.3)	0.04	1,950 (8.7)	9,644 (9.3)	0.019	0.004

SMD = standardized mean difference; n = number; BMI = body mass index; MI = myocardial infarction; COPD = chronic obstructive pulmonary disease; SNAP = supplemental nutrition assistance program; NH = non-Hispanic; AI = American Indian; AN = American Native

Supplementary Table S2. Adjusted associations between social determinants of health and prevalent diabetes in the development and validation cohorts.

Variable	Level	OR (95% CI), Development	P, Development	OR (95% CI), Validation	P, Validation
Life satisfaction	Reference: Very satisfied	1 [Reference]		1 [Reference]	
	Satisfied	1.21 (1.14 - 1.30)	<.001	1.20 (1.11 - 1.30)	<.001
	Dissatisfied	1.44 (1.23 - 1.68)	<.001	1.24 (1.06 - 1.45)	0.008
	Very dissatisfied	1.26 (1.01 - 1.58)	0.04	1.34 (1.03 - 1.75)	0.029
Emotional support received	Reference: Always	1 [Reference]		1 [Reference]	
	Usually	0.89 (0.83 - 0.96)	0.002	0.97 (0.89 - 1.06)	0.508
	Sometimes	1.00 (0.91 - 1.10)	0.976	0.88 (0.79 - 0.97)	0.01
	Rarely	0.97 (0.82 - 1.13)	0.669	0.88 (0.75 - 1.02)	0.093
	Never	1.06 (0.89 - 1.25)	0.529	0.78 (0.66 - 0.92)	0.003
Loneliness	Reference: Never	1 [Reference]		1 [Reference]	
	Rarely	0.97 (0.90 - 1.04)	0.384	0.95 (0.88 - 1.04)	0.264
	Sometimes	1.15 (1.06 - 1.24)	<.001	1.19 (1.09 - 1.30)	<.001
	Usually	1.13 (0.97 - 1.31)	0.111	1.44 (1.18 - 1.76)	<.001
	Always	1.22 (1.00 - 1.48)	0.045	1.32 (1.10 - 1.59)	0.002
SNAP receipt	Reference: No	1 [Reference]		1 [Reference]	
	Yes	1.27 (1.15 - 1.40)	<.001	1.18 (1.05 - 1.32)	0.004
Food did not last	Reference:	1 [Reference]		1 [Reference]	

	Food always lasted				
	Always	1.18 (0.99 - 1.40)	0.057	1.30 (1.10 - 1.55)	0.002
	Usually	1.46 (1.23 - 1.74)	<.001	1.34 (1.10 - 1.63)	0.004
	Sometimes	1.28 (1.14 - 1.44)	<.001	1.41 (1.24 - 1.60)	<.001
	Rarely	1.33 (1.20 - 1.47)	<.001	1.26 (1.12 - 1.42)	<.001
Transportation barrier	Reference: No	1 [Reference]		1 [Reference]	
	Yes	1.12 (0.99 - 1.26)	0.063	1.24 (1.08 - 1.41)	0.002
Utility shut-off threat	Reference: No	1 [Reference]		1 [Reference]	
	Yes	1.05 (0.93 - 1.19)	0.428	1.12 (0.97 - 1.30)	0.13
Unable to pay bills	Reference: No	1 [Reference]		1 [Reference]	
	Yes	1.11 (0.98 - 1.25)	0.096	1.08 (0.94 - 1.24)	0.252
Lost employment or hours	Reference: No	1 [Reference]		1 [Reference]	
	Yes	0.86 (0.76 - 0.97)	0.014	0.89 (0.79 - 1.01)	0.072

OR = odds ratio; CI = confidence interval

Supplementary Table S3. Subgroup performance of the full model at the fixed operating threshold in the development and validation cohorts.

Variable	Level	n 2023	Prevalence 2023, %	AUROC 2023	Sensitivity 2023	PPV 2023	CITL 2023	n 2024	Prevalence 2024, %	AUROC 2024	Sensitivity 2024	PPV 2024	CITL 2024
Age group	35 - 49	12,015	7.9	0.826	0.616	0.254	-0.05	37,065	8.5	0.829	0.64	0.269	0.012
	50 - 64	16,405	19.8	0.812	0.855	0.34	0.11	47,115	20.3	0.785	0.847	0.329	0.113
	65 - 74	14,275	26.1	0.755	0.893	0.356	0.169	41,573	25.5	0.76	0.89	0.348	0.104
	75+	11,608	25.5	0.72	0.86	0.321	0.106	35,969	24.6	0.732	0.891	0.319	0.074
Sex	Female	29,629	16.7	0.818	0.834	0.316	0.017	86,798	17	0.806	0.827	0.314	0.039
	Male	24,674	19.7	0.803	0.834	0.343	0.174	74,924	19.2	0.801	0.844	0.334	0.129
Race Ethnicity	White, NH	42,353	15.6	0.806	0.794	0.302	0.065	124,307	15.6	0.801	0.791	0.299	0.071
	Black, NH	3,952	23.3	0.792	0.911	0.349	0.033	13,381	23.8	0.778	0.908	0.339	0.013
	Asian, NH	1,137	17.7	0.824	0.744	0.333	0.341	3,132	20.4	0.831	0.816	0.414	0.407
	Hispanic	4,345	24.6	0.814	0.886	0.409	0.269	13,170	22.3	0.809	0.888	0.365	0.156
	Other, NH	2,516	17.4	0.813	0.922	0.289	-0.263	7,732	17.9	0.8	0.9	0.3	-0.175
Education	<High school	2,922	31	0.763	0.914	0.431	0.302	8,465	28	0.79	0.919	0.385	0.154
	High school	12,374	21.1	0.797	0.868	0.332	0.068	37,315	20.7	0.776	0.857	0.323	0.072
	Some college	14,647	18	0.797	0.828	0.314	0.054	43,204	19.1	0.787	0.841	0.32	0.07
	College graduate	24,224	11.8	0.817	0.732	0.276	0.051	72,332	12.2	0.82	0.743	0.287	0.069
Household income	<$25k	6,507	28	0.775	0.917	0.386	0.08	18,783	28	0.772	0.914	0.375	0.113
	$25 - 50k	11,017	23.3	0.792	0.877	0.351	0.132	32,381	22.7	0.775	0.881	0.341	0.089
	$50 - 100k	14,103	16	0.814	0.84	0.298	0.033	41,906	16.9	0.789	0.816	0.299	0.057
	>$100k	13,660	9.6	0.782	0.616	0.234	0.007	43,467	11	0.818	0.695	0.281	0.102
Health insurance	Uninsured	1,975	14	0.847	0.776	0.358	0.268	5,943	12.6	0.828	0.705	0.312	0.148
	Insured	50,916	18.3	0.807	0.834	0.327	0.092	151,735	18.3	0.802	0.84	0.324	0.082
Residence	Rural	8,232	21.6	0.805	0.855	0.359	0.177	21,969	19.7	0.792	0.868	0.325	0.051
	Urban	45,019	17.6	0.812	0.829	0.325	0.089	135,677	17.7	0.805	0.829	0.322	0.089

n = number; AUROC = area under the receiver operating characteristic curve; PPV = positive predictive value; CITL = calibration in the large; NH = non-Hispanic
